# The Laryngoscope in Medicine

**Published:** 1886-09

**Authors:** Barclay J. Baron

**Affiliations:** Physician in charge of the Throat Department, Bristol General Hospital; Demonstrator of Practical Pathology in the Bristol Medical School


THE BRISTOL

Medico-Chirurgical Journal.

SEPTEMBER, 1886.

### THE LARYNGOSCOPE IN MEDICINE.

BY

BARCLAY J. BARON, M.B. Edin.,

*Physician in charge of the Throat Department, Bristol General Hospital; Demonstrator of Practical Pathology in the Bristol Medical School.*

My object in writing the following short paper is, not to attempt to say anything new or original, but rather to repeat what has been often and well said, and once more to show how great is the help given, by an accurate use of the laryngoscope, in the diagnosis and treatment of otherwise obscure and untreatable cases.

The first man who appears to have conceived it possible to illuminate the various canals of the body, and amongst them the throat, was Bozzini of Frankfort, who, in 1807, published a work on the subject.

In 1827, Senn of Geneva tried to see the glottis of a little girl with mirrors, but failed on account of the small size of his instrument.

In 1829, Babington showed a laryngoscope—or, as he called it, a "glottiscope"—to the Hunterian Society; but he himself treated it more as a toy, than as an instrument from the use of which, good to suffering humanity was to come.

In 1836, Baumès described a mirror which he had found useful in examining the posterior nares and larynx, and was thus the first to look at the posterior nares during life.

In 1840, Liston saw œdematous growths obstructing the larynx, by means of "such a glass as is used by dentists on a long stalk," which he introduced well into the fauces.

In 1844, Avery of London invented a laryngoscope in principle very like that now in use.

Thus we see that up to this date those who had used instruments for seeing the interior of the larynx, had not made any observations of value, on the physiology or pathology of the organ.

In 1854, however, Manuel Garcia, the famous professor of singing, conceived the idea of looking at his own larynx whilst in the act of singing; and there can be no doubt that his paper, which was read by Professor Sharpey, on May 24th, 1855, before the Royal Society of London,* first showed to the world the value of the laryngeal mirror.

Garcia's observations were, as is only too often the case, treated with apathy in this country, and it was reserved for Türck of Vienna, and Czermak of Pesth, to create the art of laryngoscopy in 1857 and succeeding years.

The year 1857 must be looked on as the date of the

* "Physiological Observations on the Human Voice," *Proc. Royal Society of London*, vol. vii., No. 13, 1855.

invention of the laryngoscope, and its application in the diagnosis and treatment of disease; and thus we find that it came into use only six years after the ophthalmoscope, which was invented by Helmholtz in 1851. The mention of these dates leads me to ask the question: Why is it that, although the laryngoscope has been before the profession practically as long as the ophthalmoscope, where one of the former is used, ten of the latter are? I imagine that the only answer which can be given is, that the value of the two instruments to the general practitioner, as distinguished from the specialist, is in the same proportion as the number of them in use. If this be the opinion held, I propose to try and show that it is wrong, and that the laryngoscope cannot be omitted from the armamentarium of the physician or surgeon more suitably than the ophthalmoscope, and this entirely apart from its use by specialists.

The instrument which I find most convenient is made on the Viennese pattern, in which the reflector—which has a central hole, and which is larger than in the ordinary English laryngoscope—is held by a band fastened round the head, instead of in a spectacle frame, and can be worn with much more comfort, the weight of the reflector not pressing unpleasantly heavily on the nose; along with this are six mirrors, of various sizes, together with two handles into which they fit, a laryngeal sound, and a brush-holder, with three brushes to screw on to it.*

Having myself discovered and overcome certain difficulties in the use of the instrument, most of which are

* The whole apparatus, in a case, can be obtained of Heinrich Reiner, IX Van Swieten Gasse 10, Vienna, for about 30/-. (I mention this address, although it is that of only one of many Viennese instrument-makers, because some medical friends have had difficulty in getting the instrument.)

avoidable if care be taken, I think it may be advisable to mention them and the precautions to be taken in order to avoid them :—

1. Project the light into the fauces, so that the centre of the luminous disc corresponds with the *base* of the uvula.

2. In pulling out the patient's tongue, the observer's finger ought to be kept rather above the level of the teeth, in order that the frænum linguæ may not be injured.

3. Do not pull the tongue too far out, as reflex action is thus apt to be induced, and the tongue arches at the back and obscures the view.

4. The mirror introduced into the fauces ought to be of such a temperature as to cause no discomfort when applied to the operator's cheek. It may be heated by holding it over the lamp used in examining.

5. Be very careful to carry the mirror back into the fauces without touching the tongue, and when it is on the uvula press this gently upwards and backwards, *without rotating the mirror.*

6. Never keep the mirror in the fauces too long. Several short views may often be got without causing retching, and thus a fair estimate of the condition of the larynx obtained; whilst, if the observer tries to see everything at one introduction of the mirror, retching will ensue, and then all chance of seeing anything further at that sitting is usually at an end.

7. If the uvula is very near to the tongue, and there is thus a danger of touching the latter in introducing the mirror, tell the patient to take in a deep breath, or say "Ah," when the uvula generally rises, and then, by watching your opportunity, before it again descends, the mirror can be applied to it.

8. If the epiglottis hangs over the glottis and thus hides it, by making the patient sound a high note EE, in most cases the epiglottis rises, and allows us to get a fair view of the parts below. The mirror also ought to be introduced lower than usual, and held more perpendicularly in such a case.

9. In some patients the hyperæsthesia of the fauces is so great that, in spite of repeated attempts at examination, retching ensues immediately the mirror touches the back of the throat. For this troublesome complication many remedies have been recommended; *e.g.*, bromide of potassium has been used internally, as a gargle, and as a spray, but this I have found to be most uncertain in its action. Türck and Schrœtter paint the parts with chloroform, ether, or a solution of morphia; but that the use of the last-mentioned solution is not without considerable danger, I myself, whilst seeing Schrœtter's practice in Vienna, was witness. It was the case of a fine young soldier, from whose larynx Schrœtter proposed to remove a growth. The vocal cords and adjacent parts were freely painted by the professor's assistant with a strong morphia solution, and the man was deeply poisoned thereby, vomiting and considerable depression ensuing.

Morell Mackenzie recommends sucking small pieces of ice, which, although in many cases efficacious in numbing the faucial sensibility, has the great disadvantage of being difficult always to obtain at a moment's notice; and there is also delay and uncertainty of action.

Now, I only mention these various methods of subduing the sensitiveness of the pharynx, in order to show that they all have their drawbacks; and also in order to add my testimony to the great mass of evidence that has been of late so abundantly forthcoming, that in *cocaine*

we have the drug *par excellence* for the desired object. I have used aqueous solutions of hydrochlorate of cocaine, of strengths varying from 4 % to 8 %, and now prefer the latter concentration; and, although I have used it in a good many cases where the fauces were in a most hyperæsthetic condition, in consequence of which a thorough examination of the larynx was impossible, I have never failed to so far subdue the irritability, and that in a few minutes after freely painting the uvula, fauces, and pharynx with the solution, as to permit of a fair and, in most cases, a thoroughly satisfactory investigation, and this without any disagreeable concomitants.

The laryngoscope, as we have seen, was first shown to be of practical value, not by a physician, but by a singer; and thus, as is consonant with our ideas of scientific methods of research, a knowledge of the pathology of the vocal apparatus was preceded by that of its physiology. Until Garcia looked at his own larynx whilst the vocal cords were vibrating and producing musical notes, nothing definite was known of the way in which the structures that were found on dissection to form the various parts of the organ of speech and song acted, to produce the various sounds which it is capable of uttering. Since Garcia's time much attention has been directed to this side of the question; and who shall say to what lengths investigation may not go, when we find that Lennox Browne and Behnke have actually produced photographs of the vocal cords and adjacent parts, whilst the cords were sounding various musical tones? And thus they consider that they have added important information respecting the vexed question of voice registers. According to these investigators, there are no less than five registers,—a register consisting "of a series of tones which are produced by the same mechanism." * In each of these registers there is a visible difference, as seen by the laryngoscope, in the shape, vibrating portions, and position of the vocal cords and surrounding parts; and thus a variation in the mechanism that is set in action causes a variation in the structures that produce musical notes, and thus different notes are emitted from the organ. In order that others may see this as well as they, the aid of photography has been called in, and the image of the registers permanently retained. This has also been well done by Dr. French, who exhibited his photographs to the International Medical Congress at Copenhagen, in 1884.

Laryngoscopy, then, is of much value in physiological research; but of how much greater value is it, when we turn to what more nearly concerns us as medical men; viz., pathological investigation! I may then, perhaps, be allowed to relate, very briefly, a few of the conditions which have come under my own notice in hospital and private practice, in which I have derived much help from its employment.

1. *Foreign Bodies in the Throat.*—In these cases, those who do not use the laryngoscope are obliged to use an instrument which is very much less delicate for examining the fauces, &c.; viz., the forefinger, which may be long and thin, and fairly suitable, or short and thick, and most unsuitable for the purpose. Much groping in the dark ensues; the search may be successful or not, but as a rule the patient is submitted to a great deal of discomfort, the parts are congested and made sore, and, after all, the laryngoscope may have to be employed, but under the

* *Voice, Song, and Speech.* By Lennox Browne and Emil Behnke. 3rd Ed., pp. 163. Sampson Low & Co., London. 1884.

greatest difficulties, owing to the worried condition of the throat. As all of us know, foreign bodies in the throat scratch the parts, are distinctly felt, and then it often happens that, after violent exertions on the part of the patient, they pass down into the stomach—it may be from the fauces, or from the side of the epiglottis, or from the pharynx, or even the larynx. But, although the foreign body is safely conveyed into the stomach, *the sensation of its presence in the place where first it lodged, and where, it may be, it scratched the mucous membrane, remains;* and the patient demands its removal. The practitioner, without a moment's delay, proceeds to try and do this, and after groping about in utter darkness, with the finger rubbing against delicate and worried structures, he fails to accomplish anything useful; more pain and distress ensue, and the patient leaves the consulting-room feeling that something which is "sticking in the throat" ought to be taken away.

Foreign bodies lodge between the anterior and posterior pillars, and behind the posterior pillar of the fauces, and the finger is very useful in exploring these crypts. If, however, we find nothing in one or other of these situations, we ought to use the laryngoscope, and to look first of all at the space between the base of the tongue and the epiglottis, and then at the lower part of the cavity where the cricoid and arytenoid cartilages project posteriorly. If nothing is seen, then it may be proper to use the finger, to feel for any small sharp-pointed body which has pierced the mucous membrane; but the laryngoscope ought to be used, before rough manipulation has rendered the fauces so congested and irritable, as to make it very difficult to get a satisfactory view. The use of cocaine solution is most valuable both in enabling us to carry out a full examination, and to remove any foreign body that is found, without pain. For the relief of the disagreeable sensation of something abnormal remaining in the throat, I prescribe a 1 per cent. solution of cocaine, to be used as a gargle or as a pigment, and in addition, if there is a good deal of congestion, Glycerine of Tannic Acid, to be painted on the throat.

2. *Laryngeal Œdema.*—A few months ago a woman was admitted, under my care, at the Bristol General Hospital, with the following history:—

She had had, for a few weeks, a "bad cold," with a little cough, and the sore throat which so often accompanies such a condition, but was able to do her work, and did not lie up for it at all. On the morning of the day of her admission, soon after breakfast, swallowing became more painful, and she found that she could not breathe so freely as usual; the difficulty of breathing, at first gradually and then rapidly increased until mid-day, when she was brought to the hospital in a most dangerous condition. She was sitting up in bed, her face white, with a purplish hue of part of her cheeks, nose and lips deeply cyanotic, her eyes almost starting from her head, and perspiration standing on her forehead. Her efforts to breathe were very great, and all the extraordinary muscles of respiration were in action; she constantly coughed up thick phlegm, which contained nothing at all like false membrane; her cough was toneless, and her voice reduced to a hoarse whisper; and every now and then, after a moment's ease, there came a paroxysm of coughing, and she fairly struggled for life.

I found that the larynx and trachea were very sensitive to touch, and merely placing my fingers lightly on the skin over these parts, excited such a spasm that I thought asphyxia must have speedily ensued.

Here, then, we had to do with a most alarming case of sudden impediment to respiration, and the probable condition of things was œdema of some part of the larynx, seeing that we had neither the history, nor the brassy cough, nor the expectoration of false membrane, of croup; and on looking into the fauces and finding nothing abnormal, except a little congestion, diphtheria could be excluded. But there was only one way to make a *probable* diagnosis *certain*, and that was by examining the larynx with the laryngoscope. This I at once did, and got a very satisfactory view of what was to be seen of it. I found the epiglottis enormously swollen, its mucous membrane of a red colour and semi-transparent, and it was so curved on itself as to leave merely a small hole or gap between its lateral extremities, through which the air entered and left the lungs. None of the glottis could be seen, as the swollen epiglottis quite blocked the view. I ordered a succession of hot poultices to the larynx; had a croup-tent rigged up; kept steam constantly going into the tent, impregnated with Tinct. Benzoin. Co.; fed her with sips of hot milk and beef-tea; and Dr. Newnham, the house surgeon, kindly undertook to be ready to perform tracheotomy if these measures were not sufficient to control the swelling, and if asphyxia threatened.

Next day she was somewhat better, had brought up more than a pint of tough stringy phlegm, the epiglottis was less swollen, the gap through which breathing was performed was larger, and the posterior part of the glottis could be seen, and was quite free from swelling; the larynx and trachea were much less tender to touch, and the cough and pain on swallowing were less. Treatment the same as before. Next day the epiglottis was still less swollen, very red on the right side, and looked as if it might bleed; there was no swelling anywhere about the arytenoid cartilages, nor in the aryteno-epiglottic folds; phlegm still brought up, but not so freely; breathing easier, and swallowing less painful. Treatment as before. Day by day the epiglottis gradually returned to its normal shape and colour: the glottis thus became more and more visible, the secretion of phlegm diminished, the cough and pain on swallowing disappeared, and the breathing became normal. After staying in hospital a short time to regain her strength, she went home quite well.

I have given this rough sketch of the case in order to show that it was only by using the laryngoscope, that the *diagnosis was made certain*, and that I was able to watch the progress of the disease from day to day.

3. *Symptoms observed in Throat Disease simulating and mistaken for those found in Chest Affections.*—One case, in which the laryngoscope enabled me to accurately and easily diagnose what could not even be guessed at without its aid, shows the advantage of using the instrument.

It was that of a young woman, who came to me as an out-patient at the Bristol General Hospital; she had had a hard cough, with very little expectoration, and diminution in vocal power and clearness for many years, in fact from early childhood; she had been treated with "cough mixtures" *ad nauseam*, but, spite of all the drugs that had been administered, the cough continued day and night; and although there was no loss of flesh of any consequence arising from it, still the strain, loss of sleep, and general disturbance made her look thin and anxious.

On examining the chest, I found nothing to account for the symptoms.

What then could be the matter?

The examination of the throat fully answered the question. The fauces were congested, as one would expect them to be, seeing that there had been so much cough. The laryngoscope showed that on the middle third of one of the vocal cords there was a small growth, which, on attempted phonation, prevented the accurate and normal apposition of their inner edges, and so rendered clear voice impossible; at the same time, the vocal cord opposite the one on which the growth was situated was congested, and so the hard cough was accounted for.

The cure of such a condition is simple—viz., removal of the small growth—and cough mixtures are useless, or worse.

Another case, of a powerful young man, who came to me complaining of a constant tickling cough, accompanied by very little expectoration, and that in tiny pellets, got up after much exertion, may serve as a second illustration.

Here, the lungs were quite normal, but the fauces were congested, the tonsils somewhat enlarged, the uvula very long and trailing on the back of the tongue; and on examining with the laryngoscope, a congested condition of the mucous membrane of the larynx and trachea was seen. Insufflations of powdered alum, brushing the affected parts with a solution of chloride of zinc in glycerine, soothing inhalations, and shortening the uvula, gave very great relief; whilst cough mixtures, of which he had had many, had been proved to be of little value.

4. *Chest Affections giving rise to symptoms simulating and mistaken for those found in Throat Disease.*—Such a case as the following shows the value of laryngoscopy in this connection. A man, about 45 years of age, came to me at the Bristol General Hospital, as an out-patient, complaining of hoarseness, cough with some expectoration, and debility. The cough was like what one hears in chronic laryngitis; and, indeed, before I saw him, a medical man of great experience had diagnosed that affection. Laryngoscopy showed paralysis of one vocal cord, and on examining the chest, I found evidence of such a condition of things as would lead to pressure on one of the recurrent laryngeal nerves; and thus the symptoms were explained, and I could feel certain that they did not arise from throat disease, and, therefore, that local treatment was not needed.

5. *Throat Conditions found in Phthisis.*—Without going into the very interesting question of the tubercular throat affections which are found to accompany or to precede pulmonary phthisis, I wish to show that it is of much value to *know the exact condition of the larynx* where, in a case of pulmonary phthisis, the voice is altered, or swallowing is painful. In many such cases, we find that the laryngeal mucous membrane is simply anæmic; *i.e.*, it participates in the general anæmia; but this condition, in a certain number of these cases, precedes actual tubercular infiltration, the lowered nutrition of the parts rendering them a prey to the invasion of tubercle bacilli, from direct contact of the phthisical sputum or otherwise.

In other cases we find the epiglottis swollen, and so curled on itself that it assumes a turban-like form; as the disease advances, we get ulceration of this structure. Where this is the case, we find that the patient has much pain in swallowing, and food is apt to get into the air passages, the damaged epiglottis not covering the top of the larynx accurately, on swallowing.

In other cases the aryteno-epiglottic folds are affected.

At first they are much swollen, from infiltration with tubercular matter, and from œdema, and then there is difficulty in breathing, which is noisy and painful; *later*, ulceration of the infiltrated structures supervenes.

In other cases the vocal cords may be merely thickened; but they will become ulcerated sooner or later.

Lastly, paresis, or even paralysis, of one vocal cord supervenes. In the last two conditions, the voice becomes deep, hoarse, or toneless, and speaking is painful.

Thus, in a phthisical case, where there are throat symptoms, we may have to do with conditions of the larynx, varying from mere anæmia up to the severest forms of ulceration and paralysis, all of which are visible to the observer who uses the laryngoscope, and to him alone.

And not only is it of value for us to *diagnose* these conditions, but also to enable us to *treat* them; and by the accurate application of solutions of cocaine, or of insufflations of morphia and iodoform, we can do much to alleviate pain; and by enabling the patient to take food *painlessly*, we materially promote his general well-being.

Then, again, we may be able to diagnose phthisis laryngea even before there is any such affection of any other organ as to enable us to say that it is diseased.

As types of this state of affairs, take the following cases that have come under my care :

1. A sailor, about 30 years of age, complaining of laryngeal cough, hoarseness, night-sweats, and increasing debility. There was no affection of the lungs that I could discover; but he had an unmistakable turban-like infiltrated epiglottis, which was so placed as to prevent my getting a view of the glottis. I have lost sight of him, and do not know if the lungs ultimately became diseased; but I have little doubt as to the throat condition being phthisical.

2. A sugar refiner, about 55 years of age, whose duty it was to taste the acid material which is formed in the process of refining. This man complained of shortness of breath, especially on exertion, laryngeal cough, and alteration of voice, occasional night-sweats, no marked loss of flesh. His voice was deep and hoarse, and his cough and respiration were significant of laryngeal stenosis.

The laryngoscope revealed considerable swelling of the right aryteno-epiglottic fold and the parts immediately contiguous to it, and the symptoms of obstruction were due to the swollen mucous membrane, which partially blocked the glottis. The lungs were carefully examined, without finding any disease in them. I watched this case for some time, and saw the parts affected with infiltration gradually break down, until there was an open discharging surface larger in diameter than a pea, from which there exuded thick caseous material, the odour of which was abominable. The right lung also gradually became affected, and from being consolidated, began to show signs of breaking down. For purposes of diagnosis, and also for treatment, of such a case of primary phthisis laryngea, the laryngoscope was invaluable; and without its aid I could never have arrived at even approximate accuracy. I was also able to help him by applying remedies accurately to the diseased spot.

3. A gentleman, aged 25, was sent to me by my friend Dr. Markham Skerritt, suffering from pain on swallowing, deep hoarse voice, cough which was almost toneless and accompanied by expectoration, and increasing debility.

His history showed that some years ago Dr. Ord told him that he had some ulceration of the throat, but that his lungs were quite sound. His voice and cough were almost exactly like those of the preceding case: there was much pain on swallowing, increasing debility, and occasional night-sweats. Laryngoscopy showed that the epiglottis was much thickened and deformed, and ulcerated along its upper edge in several places, which were discharging; there was some swelling of the mucous membrane of the sides of the larynx, and also of the aryteno-epiglottic folds; the vocal cords were ulcerated, and had out-growths on their edges. All these appearances pointed infallibly to laryngeal phthisis; and the examination of the lungs showed mischief at both apices.

Cure was out of the question; but by applying, with the aid of the mirror, iodoform, and other antiseptics, to the ulcers, they took on a more healthy action. His voice improved and cough lessened; and by painting the epiglottis with a solution of cocaine, he was able to take his food with comfort, and thus ate more, and improved in consequence.

As a contrast to the above case, where there was much need of local throat treatment, I may relate that of a patient in whom there was manifest evidence of phthisis of both pulmonary apices, and who had alteration of voice and almost toneless cough accompanying it. Here the laryngoscope showed that one vocal cord was paralysed; but that there was no local throat disease, the chest condition causing the paralysis by interference with one of the recurrent laryngeal nerves. In such a case, it is apparent that the throat needed no local treatment.

6. *Throat Conditions found in Syphilis.*—In this disease, in the vast majority of cases, the upper part of the throat is alone affected, and the laryngoscope is not required, but there is a certain small proportion of cases in which secondary and tertiary syphilis attacks the epiglottis and larynx, and in these the instrument, for purposes of diagnosis and treatment, is of great value. It is, perhaps, of still greater value in enabling us to decide between laryngeal stenosis, due to phthisical infiltration of parts, and malignant or syphilitic new growth, such as we see in the tertiary stage of the disease.

